# Activation energy of aluminate dissolution in metakaolin: MLFF-accelerated DFT study of vdW and hydration shell effects

**DOI:** 10.1039/d5na00103j

**Published:** 2025-06-03

**Authors:** Mohammadreza Izadifar, Neven Ukrainczyk, Klara Schönfeld, Eduardus Koenders

**Affiliations:** a Institute of Construction and Building Materials, Technical University of Darmstadt Franziska-Braun-Str. 3 Darmstadt 64287 Germany mr.izadifar@icloud.com izadifar@wib.tu-darmstadt.de

## Abstract

This research utilizes computational chemistry to investigate the complex mechanisms driving the dissolution of thermally activated metakaolin (MK) clay, a key supplementary cementitious material (SCM) in the manufacturing of concrete and geopolymer-based materials, thereby contributing to a reduced carbon footprint. A thorough exploration of the dissolution process is fundamental for fully understanding its pozzolanic reactivity. Expanding on our recent investigations into SiO_4_^4−^ dissolution in MK, this work addresses critical data gaps in understanding the dissolution behavior of aluminate species. The findings complement essential input for microscopic forward dissolution rate computations using the atomistic kinetic Monte Carlo (kMC) upscaling approach. To this end, the study calculates the atomistic activation energy (Δ*E*_a_) of aluminate species at the transition state for the hydrolysis reaction using machine learning force fields (MLFF) based on density functional theory (DFT) and the improved dimer method (IDM) under far-from-equilibrium conditions, focusing on three activators: NaOH, KOH, and water. The analysis explores both the presence and absence of van der Waals (vdW) interactions, along with varying geometric configurations of hydration shells surrounding cations (Na^+^, K^+^) and the hydroxide anion (OH^−^). The findings indicate that KOH generally exhibits lower Δ*E*_a_ than NaOH, especially when vdW interactions are considered. Moreover, the findings emphasize that reduced hydration shells around KOH and NaOH lead to lower Δ*E*_a_ for the dissolution of aluminate species.

## Abstract 2

This research focused on calculating the atomistic activation energy (Δ*E*_a_) for aluminate dissolution using MLFF based on DFT, applying the improved dimer method (IDM) under far-from-equilibrium conditions.

## Graphical abstract

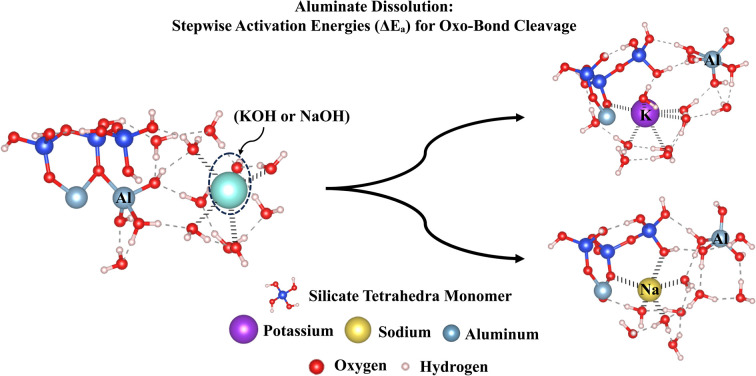

## 1. Introduction

MK is the key component of calcined clays due to its pozzolanic reactivity and is widely utilized in various applications within the construction industry.^1–3^ MK (calcined) clay is derived from dehydroxylation (DHX) of kaolinite by heating it to temperatures between 400 and 700 °C.^4^ In other words, the primary mineral phase, known as “ideal or ordered kaolinite” (Al_2_(OH)_4_Si_2_O_5_), undergoes dehydroxylation to form MK (Al_2_Si_2_O_7_) at temperatures between 500–700 °C. In contrast, “low-crystallinity” or “disordered kaolinites” which have stacking faults, dehydroxylate into metadiskaolinite at lower temperatures ranging from 400–700 °C. Complete dehydroxylation at stationary points leads to an expansion of around 4% for MK supercells and approximately 8% for metadiskaolinite supercells, compared to their ideal and disordered kaolinite counterparts, as demonstrated in the study by Izadifar *et al.*^4^ Kaolinite calcination produces millions of tons of MK annually, which is utilized as a SCM in the production of concrete and geopolymer-based materials.^5^ Replacing up to 30% of the cement by weight with MK typically results in concrete that is more cohesive with reduced bleeding, enhanced compressive strength,^6,7^ lower CO_2_ emissions,^8^ and decreased porosity.^7^ Additionally, it offers improved resistance to attacks from sulfates, chlorides, and other aggressive agents,^9^ including minerals and organic acids.^10–12^ Meta-clay materials and industrial byproducts, which are rich in reactive silica and alumina, exhibit reactivity that is primarily governed by their dissolution rates. This makes them ideal for improving ecological efficiency by optimizing cement replacement and producing geopolymers,^13,14^ leading to a reduced carbon footprint. To fully harness their potential, atomistic simulation techniques will be employed to investigate the dissolution processes of these materials, providing crucial insights into their reactivity and facilitating the development of more effective formulations for sustainable construction applications.

Atomistic simulation techniques have recently been essential in uncovering the microstructure of materials like cement clinker,^15,16^ hydrated cement phases,^17,18^ and glasses,^19^ as well as the connection between atomistic configurations and their reactivity.^20,21^ Approaches such as density functional theory (DFT),^22^ molecular dynamics (MD),^23^ and *ab initio* molecular dynamics^24,25^ computational methods are widely employed in chemistry and materials science to study reaction mechanisms, mechanical behavior,^26,27^ and other material properties. Coopamootoo and Masoero^28^ examined the dissolution of tricalcium silicate at screw dislocations within finite grains, considering various facet orientation combinations but excluding crystallographic defects. Their study revealed that dissolution proceeds by consuming kink particles in a sequential, layer-by-layer fashion, initiating at low-coordinated sites located at the intersections of facets, such as edges and corners. These sites were found to have comparable dissolution rates. Furthermore, the dissolution rates of defect-free, finite-sized crystals showed a linear relationship with β, aligning with the principles of traditional transition state theory (TST). N'Guessan *et al.*^29^ investigated the leaching of Al and silicon in various alkali hydroxide solutions. They found that the dissolution of kaolinites was influenced by the concentration of hydroxides, with Na^+^ promoting greater dissociation than K^+^ due to its higher charge density. Briki *et al.*^30^ observed that the reaction of slag slowed down during both the initial and final stages. In the first 30 minutes of exposure to a slag-NaOH solution, they identified a non-steady state dissolution phase for Al, Si, and sulfur ions, which was followed by a steady state and then a decline in the release rates of Al and silicon due to precipitation. Chen *et al.*^31^ developed an integrated dissolution model for alite (tricalcium silicate)^32^ to examine the effects of varying hydrodynamic conditions on dissolution rates, from low to high. The model combined surface topography with ion transport using the kMC approach, effectively addressing both dilute and near-saturated conditions. Their simulation outcomes were largely consistent with existing literature. Valentini^33^ also reported modeling dissolution-precipitation kinetics of MK using the cellular automaton reaction-diffusion model HydratiCA. Moreover, Izadifar *et al.*^16,18^ investigated the microscopic forward dissolution rates of β-C_2_S cement clinker, and portlandite under far-from-equilibrium conditions through atomistic kMC^16,18,34^ upscaling approach, utilizing atomistic Δ*E*_a_ derived from MetaD molecular dynamics (MD) simulations methods.^15,17,35^ Most recently, Izadifar *et al.*^36^ analyzed the nucleation mechanisms of alkaline aluminosilicate gels, including MK, by examining the binding energies (Δ*G*_reaction_) of four distinct monomer species through a coarse-grained Monte Carlo (CGMC) approach. Valencia *et al.*^37^ also employed CGMC simulations, utilizing octree cells to investigate the nucleation of geopolymers at various pH levels.

In VASP, the IDM by Heyden *et al.*,^38^ is used to optimize the transition states. This approach determines the minimum energy pathway, tracking atomic movements between initial and final states. The Δ*E*_a_ is identified by maximizing the potential energy along the unstable mode or decay direction (dimer axis), while energy minimization occurs in all other directions. Although there has been significant research on clays, few atomistic computational studies have been reported that specifically examine the reactivity of protonated silicate tetrahedra in MK (calcined) clay, while considering the effects of neighboring aluminate and silicate units. Izadifar *et al.*^20^ applied the IDM and transition-state theory (TST) within the framework of DFT to calculate the atomistic Δ*E*_a_ for the dissolution of MK silicate tetrahedra by water molecules, excluding the effects of vdW interactions under far-from-equilibrium conditions. They found that the Δ*E*_a_ needed to break the oxo-bridging bond with a neighboring silicate unit was higher than that for an aluminate unit, attributed to ionic interactions. Most recently, Izadifar *et al.*^21^ used the IDM to explore how reaction enthalpy evolves during the hydrolysis and breakdown of silicate tetrahedra (SiO_4_)^4−^ including water as an activator in MK under far-from-equilibrium conditions. The computations, based on TST, aimed to determine the Δ*E*_a_ for (SiO_4_)^4−^ dissolution in MK, incorporating two activators of NaOH and KOH with and without contribution of vdW interaction and hydration shell around cations. Cheng *et al.*^39^ also utilized the dimer method to locate transition states, which were verified to have a single imaginary vibrational frequency through DFT calculations.

Comprehending the dissolution mechanism of MK at the macro scale is essential for enhancing its application as a supplementary cementitious material and as a cement-free geopolymer binder. Additionally, it is important to note that the Δ*E*_a_ for aluminate species dissolution is currently lacking in atomistic simulations. The primary objective of this study is to calculate the atomistic Δ*E*_a_ at the transition state for the hydrolysis reaction using MLFF^40^ based on DFT through the IDM to determine the change in reaction enthalpy under far-from-equilibrium conditions. The computations, based on Transition State Theory (TST), focus on determining the atomistic Δ*E*_a_ for the dissolution of aluminate species by formation of aluminum hydroxide hydrate Al(OH)_3_(H_2_O)_3_, in MK, considering three activators, NaOH, KOH, and water. The study examines scenarios both with and without the contributions of vdW interactions and various geometry hydration shells around cations (Na^+^ and K^+^) and the hydroxide anion (OH)^−^. Additionally, to enable comparisons of the calculated (Δ*E*_a_) values derived from high-alkaline environments (NaOH and KOH), the study also includes computations of (Δ*E*_a_) for the hydrolysis reaction in the presence of water, considering both the inclusion and exclusion of vdW interactions. In addition to our previous observations on SiO_4_^4−^ dissolution in MK,^20,21^ the findings from this study address the existing data gaps vital for 6-fold coordination (Al^VI^) dissolution of aluminate species, preparing all the input data for performing microscopic forward dissolution rate calculations, which are crucial for the atomistic kMC upscaling approach.

## 2. Materials and computational methods

### 2.1. Materials

**Fig. 1:**
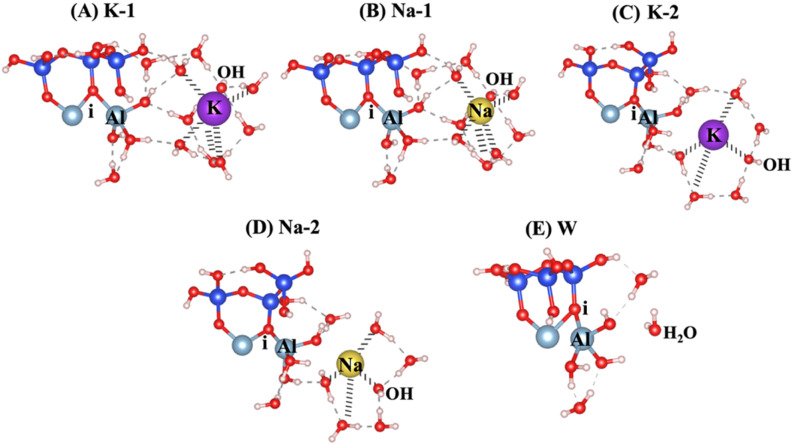
Model scenarios for dissolution of aluminate species through different activators of (A and C) potassium hydroxide (KOH), (B and D) sodium hydroxide (NaOH), and (E) water (H_2_O). In scenarios (A) and (B), the cations (K^+^ and Na^+^) exhibit octahedral coordination, while the hydroxide anions (OH^−^) adopt tetrahedral coordination. In scenarios (C) and (D), the cations exhibit tetrahedral coordination, and the hydroxide anions adopt trigonal planar coordination. Silicon ions are shown in blue; aluminum cations, in light blue; oxygen ions, in red; hydrogen protons, in white; sodium cations, in yellow; potassium cations, in purple.

**Fig. 2:**
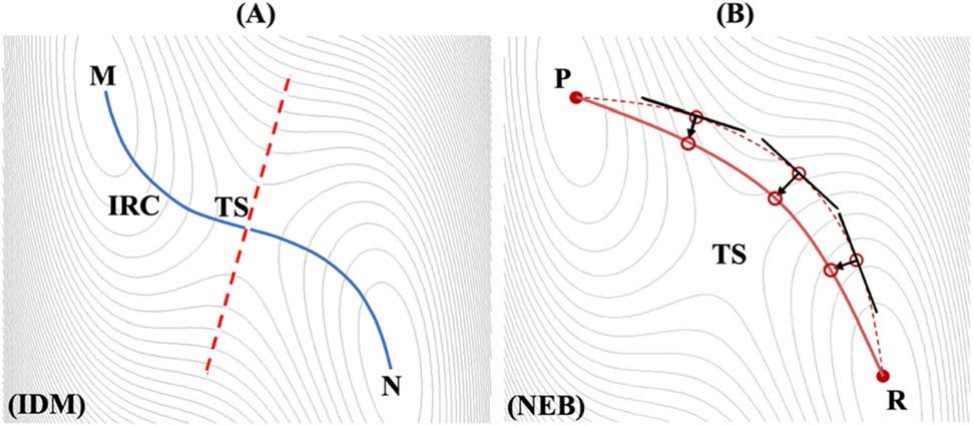
Methods for transition state identification along the reaction pathway (A) improved dimer method (IDM)^38,53^ representing intrinsic reaction coordinates (IRC) between M and N (B) nudged elastic band (NEB) highlighting minimum energy path between R and P.^51,52^

The surface energies (*γ*) for low Miller index facets were determined for three different surface orientations: 001, 010, and 100, as detailed in recent study.^21^ The calculated values were 0.0057, 0.116, and 0.18 eV Å^−1^ for the 001, 100, and 010 orientations, respectively. These results reveal that the 001 or 001̄ surface orientation exhibits the highest exposure and reactivity among the surfaces in clay-derived materials like MK. The structure of MK corresponds to the 001 and 001̄ surfaces, and simulations have been conducted to study the dissolution of Al^VI^ from 001̄ surface that include breaking of the ionic bond from the neighboring oxygen (i) attached to adjacent silicate unit, as shown in Fig. 1. An overview of the model scenarios, including activators, their abbreviations, and the corresponding hydration shell configurations, is presented in Table 1. The dissolution of Al on the MK 001̄ surface depends on the type of activator used and the varying number of water molecules with hydroxide in hydration shells surrounding the activators. The hydration shell configurations and number of water molecules were selected based on well-established coordination chemistry principles and are supported by prior studies.^41^ Thus, in order to make a comprehensive investigations, we modeled sixfold and fourfold hydration shells around potassium (K^+^) and sodium (Na^+^), and three to fourfold hydration around hydroxide anions (OH^−^). Fig. 1A–E (corresponding to models 1–5) presents a variety of potential scenarios for aluminate species dissolution, emphasizing the effects of different activators and hydration shells, including KOH, NaOH, and H_2_O. In Fig. 1A (K-1) and B (Na-1), the potassium (K^+^) and sodium (Na^+^) cations exhibit octahedral coordination, while the hydroxide anions (OH^−^) are tetrahedrally coordinated. In Fig. 1C (K-2) and D (Na-2), the cations are arranged in a tetrahedral coordination, while the hydroxide anions show a trigonal planar coordination. Fig. 1E (W) also shows the initial structure of reactant, including a water molecule as the absorbent on the MK surface.

**Table 1:** Overview of model scenarios with different activators, corresponding hydration shell configurations, and abbreviated name (based on Fig. 1). In the hydration shell configuration, coordination numbers of six, four, and three around cations and hydroxide anions correspond to octahedral, tetrahedral, and trigonal planar geometries, respectively. Note that the first number in each cell refers to the activating cation (K^+^ or Na^+^), and the second number refers to the hydroxide anion (OH^−^)

	(A)	(B)	(C)	(D)	(E)
Scenario (model)	1	2	3	4	5
Activator	KOH	NaOH	KOH	NaOH	Water
Hydration shell configuration	6–4	6–4	4–3	4–3	—
Number of water molecules in the shell	5–3	5–3	3–2	3–2	—
Abbreviated name (Fig. 1)	K-1	Na-1	K-2	Na-2	W

### 2.2. Computational methods

The density functional theory (DFT)^42^ quantum mechanical modeling approach was employed *via* the Vienna *Ab initio* Simulation Package (VASP).^43–46^ Electron–ion interactions were defined using the projector augmented wave (PAW) approach and pseudopotentials.^47^ For correlation exchange and correlation functionals, the Perdew–Burke–Ernzerhof (PBE) parameterization within the generalized gradient approximation (GGA) was selected.^48^ The Brillouin zone sampling was conducted by a well-converged *k*-sampling equivalent to a 1 × 1 × 1 Monkhorst–Pack *k*-points mech-size for the entire system.^49^ Structural relaxations used a plane-wave cutoff energy of 400 eV, with electronic self-consistency cycles converged to 1 × 10^−4^ eV and ionic forces relaxed below 1 × 10^−3^ eV Å^−1^. Interactions of vdW were incorporated *via* the Grimme DFT-D3 dispersion correction with zero-damping, a widely used method known for its balance between computational efficiency and accuracy across a range of molecular and condensed-phase systems. Structural model analyses were visualized with the three-dimensional VESTA software.^50^Fig. 2A and B illustrate two distinct approaches of IDM^38^ and nudged elastic band (NEB)^51,52^ for computation of the transition state (TS) along the reaction pathways between M and N, and P and R, respectively. In VASP and for this study, modified version of the original dimer method,^53^ proposed by Heyden *et al.*,^38^ is implemented, resulting in a reduction in the number of gradient calculations per cycle from six to four or three gradients along with one energy calculation, thereby enhancing algorithm performance. As shown in Fig. 2A, the TS at the saddle point has been determined through computation of the most negative vibrational mode of the trail structure, diagonalized Hessian matrix and identify eigenvector with negative eigenvalue indicating the desired transformation (trail dimer axis) and finally potential energy maximization along dimer axis and minimization along all other directions between two minima of M and N the reaction pathway. Conversely, interpolation method like NEB, indicates multiple configurations (images) distributed along the path between P and R and connected *via* harmonic springs, resulting slow convergence near the minimum-energy pathway as shown in Fig. 2B. The unstable direction is estimated locally for each image (empty circle) as a tangent to the chain connecting the images and the stable states. For each image, a relaxation step using *ab initio* forces orthogonal to (and harmonic forces parallel with) the local tangent is performed. Although more images provide a better representation of the chain, the calculations become more expensive, still less accurate than IDM, and defining the initial position of the images is not always straightforward, particularly in the case of transformations involving curvilinear atomic motion.^51,52^

**Fig. 3:**
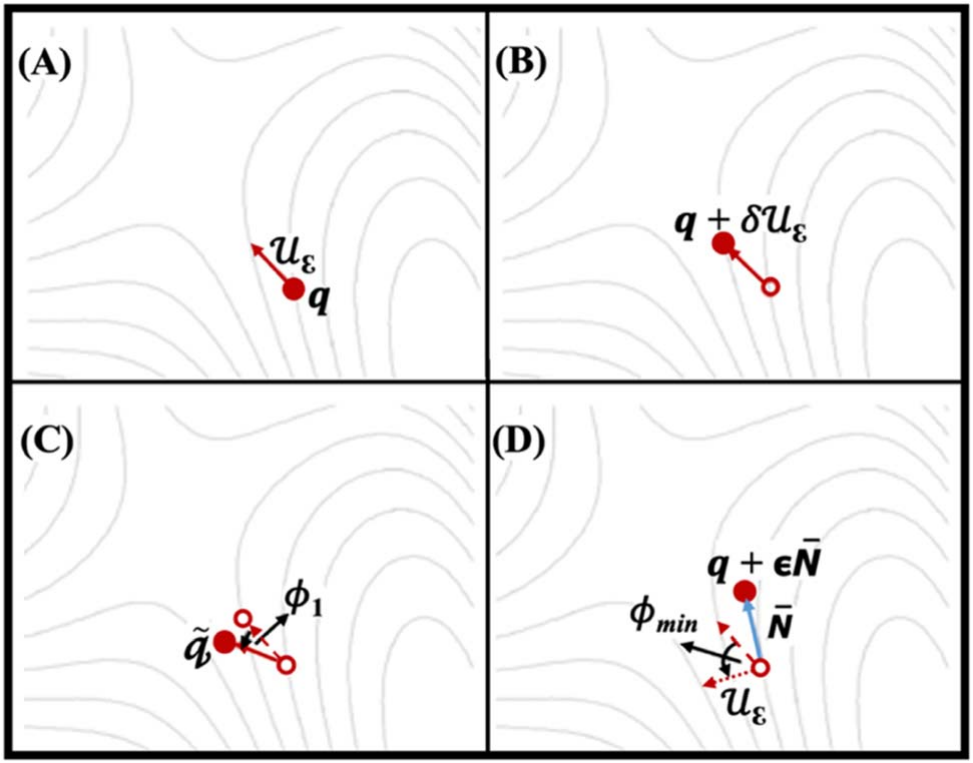
The IDM process relaxes on the potential energy surface (PES) over four ionic steps. Solid arrows represent the dimer axis (*u*_ε_), while solid circles denote the structures where forces are computed at each step. Empty circles and dashed lines indicate the structures and dimer axes from preceding steps, respectively. The dotted arrow illustrates the dimer axis after rotation by *ϕ*_min_.^38^

Fig. 3 shows four SCF calculations of force, implemented for relaxation of IDM on the potential energy surface (PES). Fig. 3A indicates the trail structure *q* and the trail initial direction (dimer axis, *u*_ε_) is taken from the most negative vibrational mode of the trail structure (*q*, using finite differences method). Fig. 3B shows an additional point on the PES forward along the trial direction is defined *q* + *δu*_ε_. The first and second points together define the dimer. Fig. 3C and D show the rotation of dimer on the PES about *q* by angle *ϕ*_1_ to maximize the negative curvature and a new direction is defined by rotating *u*_ε_ by *ϕ*_min_ to minimize the negative curvature of the PES *λ*, respectively. The potential energy is maximized along the unstable direction, (*i.e.*, dimer axis) while it is minimized in all other directions. Rotation followed by translation is followed iteratively until convergence, *i.e.* the saddle point, is reached.^38^ It is essential to note that the curvature along dimer axis must be negative during dimer method calculation. In other words, long sequence of positive values indicates that the algorithm has failed to converge to the correct transition state. In such cases, it is necessary to start with a new geometry to recompute the curvature.

A way to greatly reduce computational cost is by machine learning.^54,55^ Here, in the prediction of the target property, the method automatically interpolates between known training systems. Nevertheless, there is still the problem of how to choose the proper (minimal) training data. One very efficient and automatic way to solve that is to adapt on-the-fly learning. On-the-fly training base on the *ab initio* calculations is employed as the training set for MLFF.^40,56^ During the early steps of the simulations, specifically the first 20–30 ionic relaxation steps, the forces, energies, and stress tensors from DFT calculations are collected and stored. This data serves as the training set for the MLFF. For each new atomic configuration, VASP will check how well the MLFF predictions match the DFT-calculated forces. If the predictions are accurate (*i.e.*, within a certain threshold defined by ML_CTIFORCE), the MLFF predictions are used for the next ionic step. We set the force deviation threshold (ML_CTIFORCE) to 0.002 eV Å^−1^, meaning a DFT calculation was triggered whenever the predicted forces deviated beyond this value. If the MLFF's predictions deviate significantly from DFT, VASP triggers another full DFT calculation. The new DFT data is then used to update and improve the MLFF.

## 3. Results

### 3.1 Activation energy computation incorporating vdW contributions

**Fig. 4:**
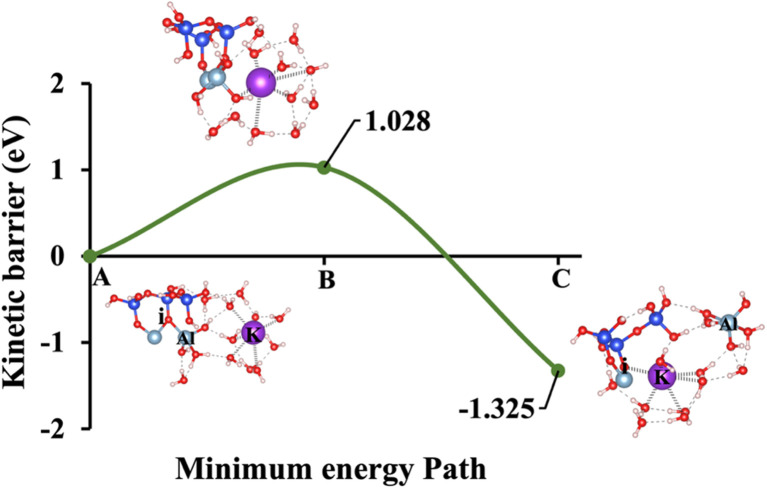
Model 1: (A) the optimized geometric structure of K-1 (illustrated in Fig. 1A) showing the breaking of the oxygen atom (i), which is bonded to the neighboring Al cation through an ionic bond with the contribution of vdW interactions. (B) The transition state was identified at the saddle point using the IDM. (C) The optimized geometric structure of the product after fully saturation of the oxygen (i) with a potassium cation (ionic bond). Energy barrier of hydrolysis reaction (Δ*E*_a_), and binding energy (Δ*G*_reaction_) obtained from first-principles calculations.

**Fig. 5:**
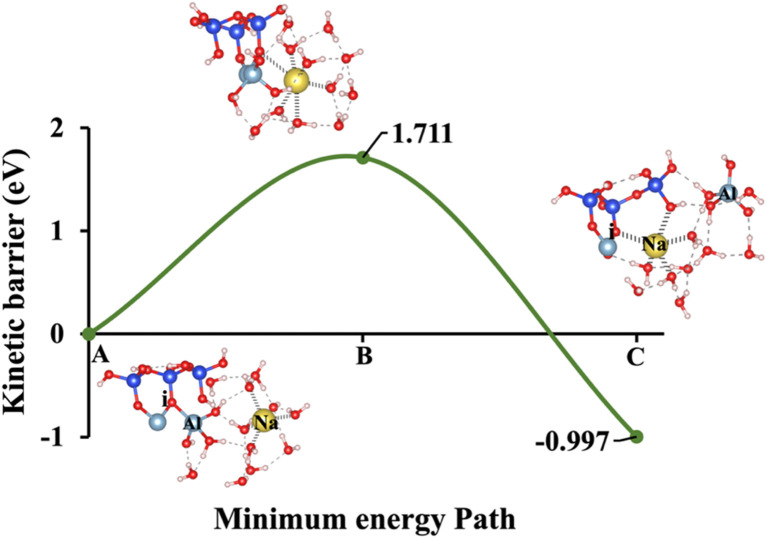
Model 2: (A) the optimized geometric structure of Na-1 (illustrated in Fig. 1B) showing the breaking of the oxygen atom (i), which is bonded to the neighboring Al cation through an ionic bond with the contribution of vdW interactions. (B) The transition state was identified at the saddle point using the IDM. (C) The optimized geometric structure of the product after fully saturation of the oxygen (i) with a sodium cation (ionic bond).

**Fig. 6:**
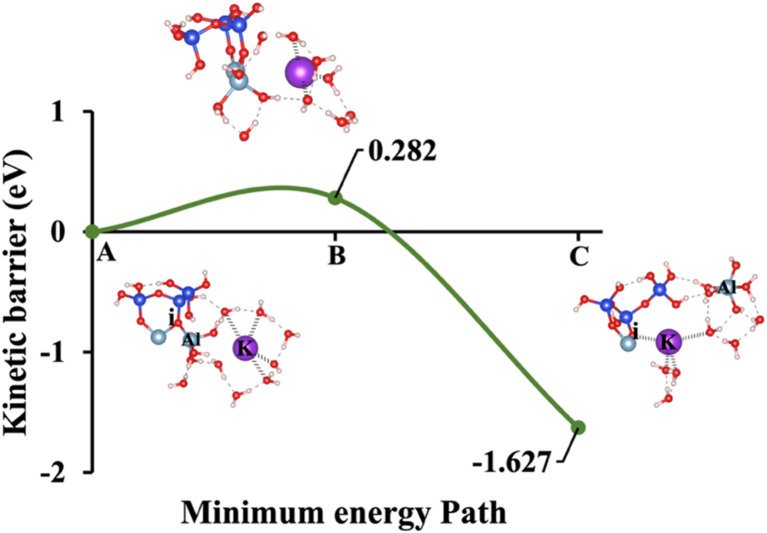
Model 3: (A) the optimized geometric structure of K-2 (illustrated in Fig. 1C) showing the breaking of the oxygen atom (i), which is bonded to the neighboring Al cation through an ionic bond with the contribution of vdW interactions. (B) The transition state was identified at the saddle point using the IDM. (C) The optimized geometric structure of the product after fully saturation of the oxygen (i) with a potassium cation (ionic bond).

**Fig. 7:**
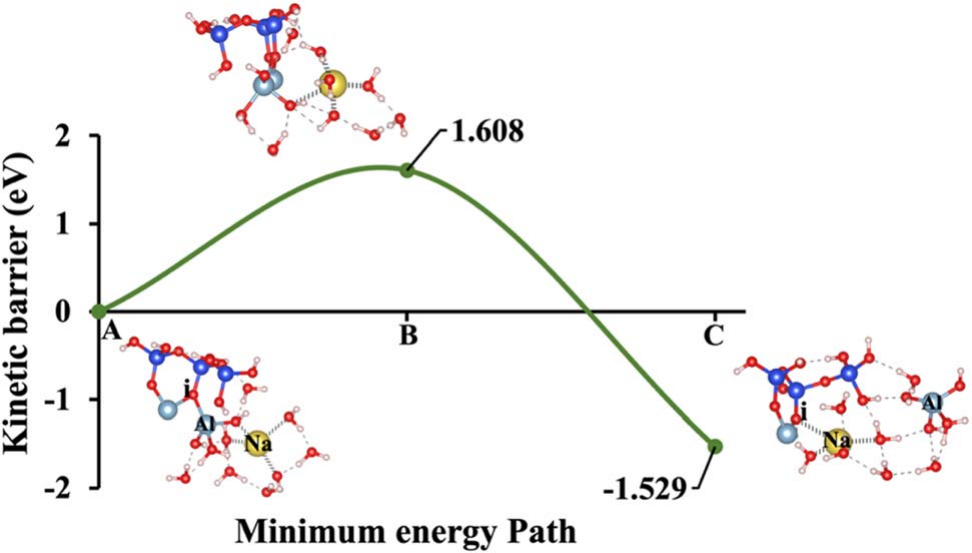
Model 4: (A) the optimized geometric structure of Na-2 (illustrated in Fig. 1D) showing the breaking of the oxygen atom (i), which is bonded to the neighboring Al cation through an ionic bond with the contribution of vdW interactions. (B) The transition state was identified at the saddle point using the IDM. (C) The optimized geometric structure of the product after fully saturation of the oxygen (i) with a sodium cation (ionic bond).

**Fig. 8:**
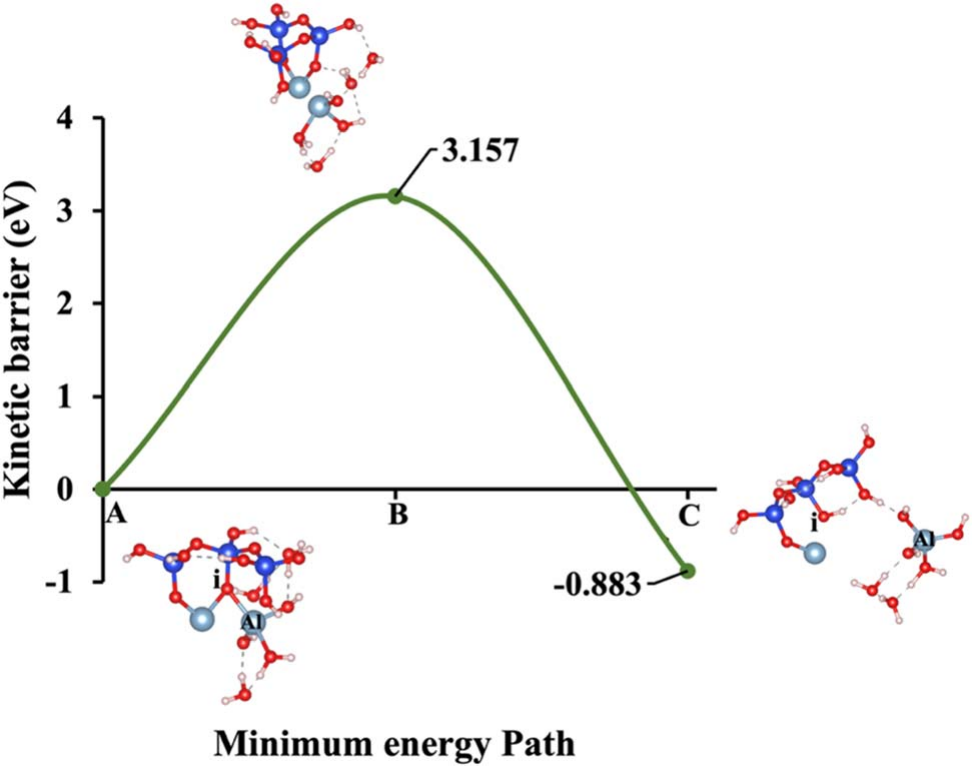
Model 5: (A) the optimized geometric structure of W (illustrated in Fig. 1E) showing the breaking of the oxygen atom (i), which is bonded to the neighboring Al cation through an ionic bond with the contribution of vdW interactions. (B) The transition state was identified at the saddle point using the IDM. (C) The optimized geometric structure of the product after fully saturation of the oxygen (i) with a proton (covalent bond).

**Fig. 9:**
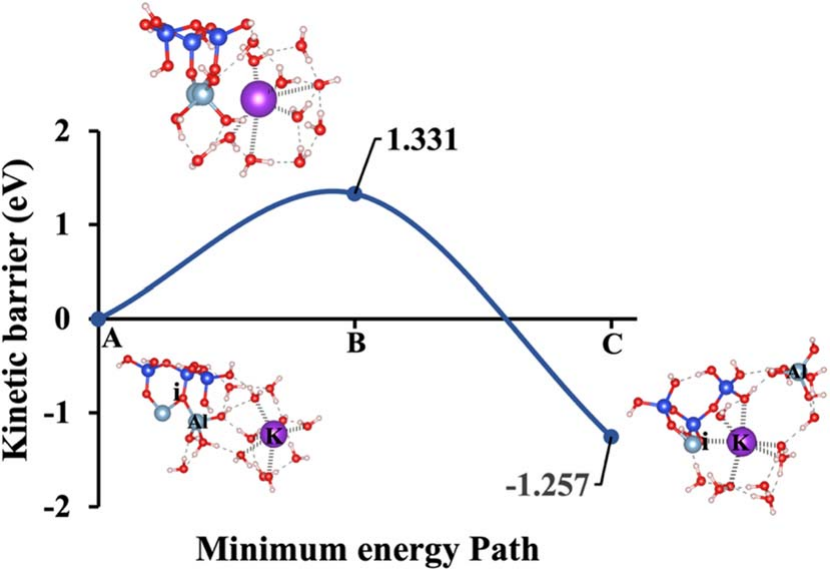
Model 1: (A) the optimized geometric structure of K-1 (illustrated in Fig. 1A) showing the breaking of the oxygen atom (i), which is bonded to the neighboring Al cation through an ionic bond without the contribution of vdW interactions. (B) The transition state was identified at the saddle point using the IDM. (C) The optimized geometric structure of the product after fully saturation of the oxygen (i) with a potassium cation (ionic bond).

**Fig. 10:**
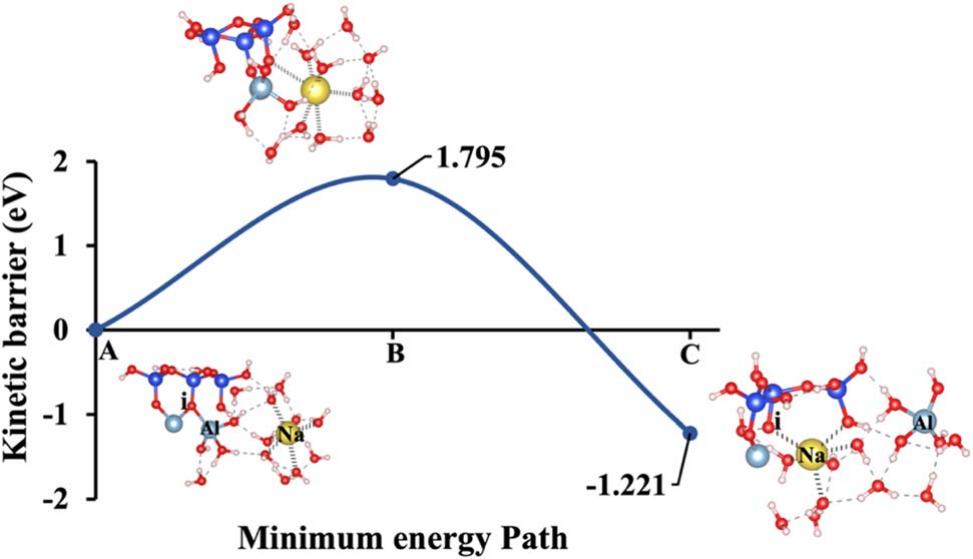
Model 2: (A) the optimized geometric structure of Na-1 (illustrated in Fig. 1B) showing the breaking of the oxygen atom (i), which is bonded to the neighboring Al cation through an ionic bond without the contribution of vdW interactions. (B) The transition state was identified at the saddle point using the IDM. (C) The optimized geometric structure of the product after fully saturation of the oxygen (i) with a sodium cation (ionic bond).

**Fig. 11:**
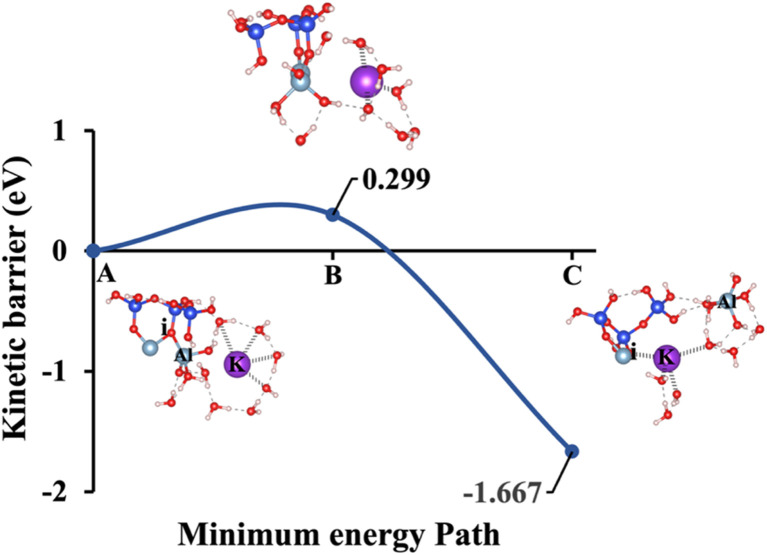
Model 3: (A) the optimized geometric structure of K-2 (illustrated in Fig. 1C) showing the breaking of the oxygen atom (i), which is bonded to the neighboring Al cation through an ionic bond without the contribution of vdW interactions. (B) The transition state was identified at the saddle point using the IDM. (C) The optimized geometric structure of the product after fully saturation of the oxygen (i) with a potassium cation (ionic bond).

**Fig. 12:**
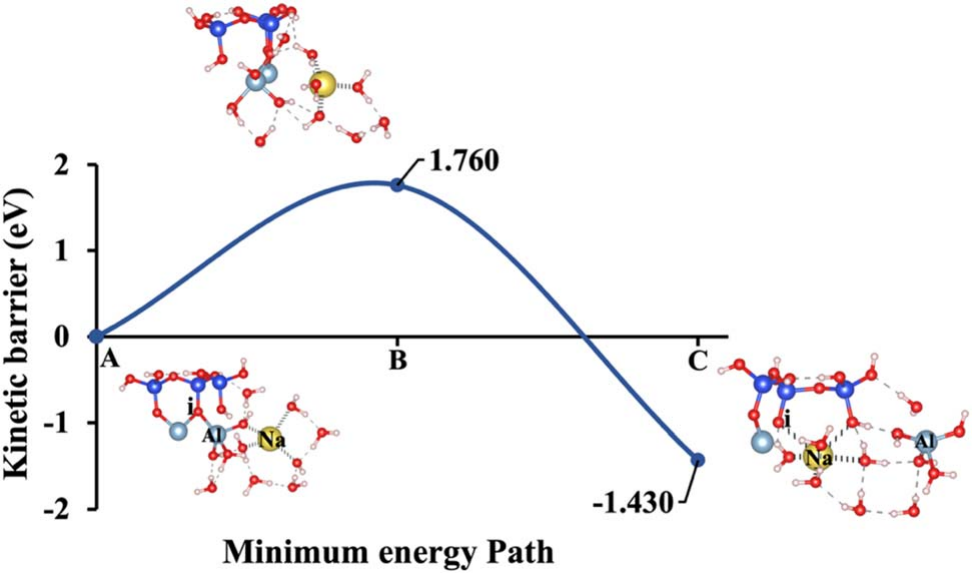
Model 4: (A) the optimized geometric structure of Na-2 (illustrated in Fig. 1D) showing the breaking of the oxygen atom (i), which is bonded to the neighboring Al cation through an ionic bond without the contribution of vdW interactions. (B) The transition state was identified at the saddle point using the IDM. (C) The optimized geometric structure of the product after fully saturation of the oxygen (i) with a sodium cation (ionic bond).

**Fig. 13:**
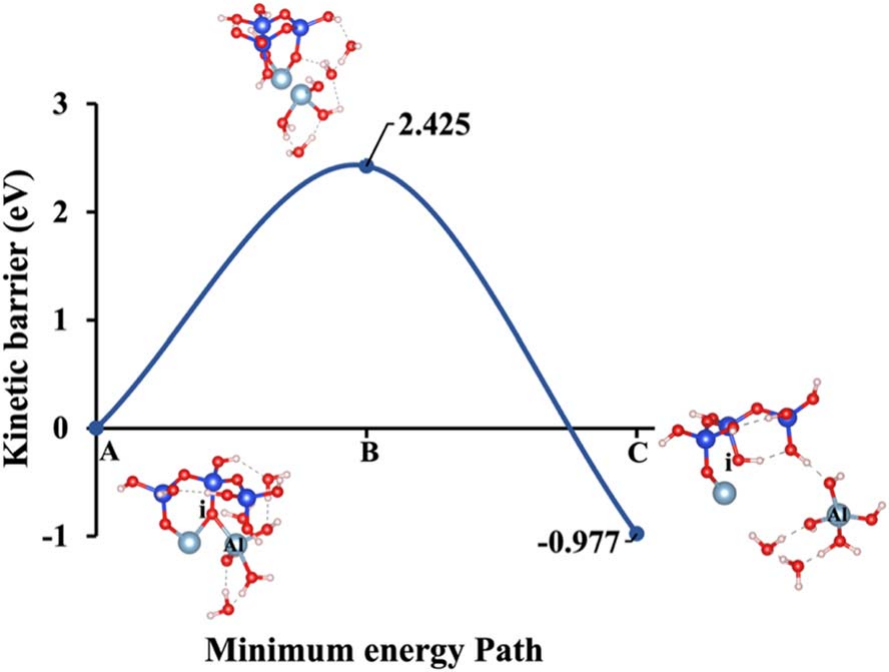
Model 5: (A) the optimized geometric structure of W (illustrated in Fig. 1E) showing the breaking of the oxygen atom (i), which is bonded to the neighboring Al cation through an ionic bond without the contribution of vdW interactions. (B) The transition state was identified at the saddle point using the IDM. (C) The optimized geometric structure of the product after fully saturation of the oxygen (i) with a proton (covalent bond).

**Fig. 14:**
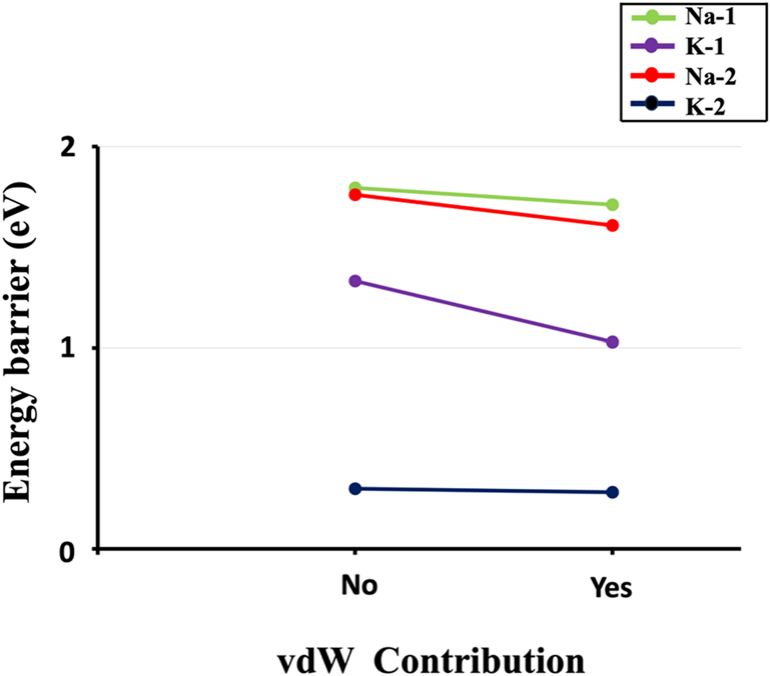
Δ*E*_a_ for aluminate species dissolutions. Dissolution of aluminate species in the presence of different activators (KOH, NaOH), as shown (labeled) in Fig. 1. The computations considered through various hydration shell around cations and hydroxide anions, with and without the inclusion of vdW interaction.

Our study aims to determine the activation energy at the saddle point using the IDM, which converges more efficiently than the NEB. While NEB maps the minimum energy path, it often struggles with convergence and requires multiple images, leading to computational inefficiencies. IDM, in contrast, directly locates the saddle point without predefined reaction coordinates, offering a more precise and cost-effective approach. To provide a comprehensive analysis, the dissolution behavior of aluminate species has been computed under 5 different models of K-1, Na-1, K-2, Na-2, and W, as depicted in Fig. 1. These scenarios encompass a range of conditions designed to capture the complexity of aluminate species dissolution processes. Specifically, the energy barrier (activation energy, Δ*E*_a_) for the hydrolysis reaction during the dissolution of AlO(OH)_2_(H_2_O)_3_ in MK has been evaluated. This evaluation considers different hydration shell geometry surrounding three activators NaOH, KOH, and water both with and without the inclusion of vdW interactions, as presented in Fig. 4–13. To enable a meaningful comparison, Fig. 14 and Table 2 juxtaposes our findings on the dissolution of aluminate species in MK with the dissolution mechanism of SiO_4_^4−^ reported in recent publication,^21^ respectively. This comparison provides the differences in dissolution kinetics, the Δ*E*_a_ profiles, and the influence of structural and chemical environments for aluminate and silicon-based species. The first scenario, referred to as model 1 and illustrated in Fig. 4, incorporates KOH as the absorbent, accounting for the contribution of vdW interactions. The initial optimized structure (Fig. 4A), which features KOH as the activator, reveals a potassium cation in an octahedral coordination (6-fold coordination), positioned 6.73 Å away from oxygen (i), while the hydroxide anions (OH^−^) are tetrahedrally coordinated (4-fold coordination).

**Table 2:** Reported SiO_4_^4−^ dissolution for four distinct scenarios (detailed in Table 1 from our recent study) under the influence of KOH and NaOH, with and without vdW interaction^21^

Model	A0S1			
Activator	KOH	NaOH	KOH	NaOH
vdW interaction	Yes	Yes	No	No
Hydration shell	Yes	Yes	Yes	Yes
Activation energy (eV)	1.462	1.949	0.788	3.276

At the transition state (Fig. 4B), the potassium cation approaches the oxygen (i) more closely within 2.76 Å, and the product's optimized geometric structure through exothermic reaction is achieved as potassium get closer through ionic reaction with oxygen (i) within 2.61 Å, resulting Al^VI^ formation. The initial optimized structure, referred to as model 2, incorporates NaOH as the activator, is shown in Fig. 5A. In this configuration, the sodium cation exhibits octahedral coordination and is positioned 6.01 Å away from oxygen (i). At the transition state (Fig. 5B), the sodium cation is located at a distance of 2.85 Å. In the optimized geometric structure of the product (Fig. 5C), resulting from the ionic reaction, oxygen (i) is positioned 2.61 Å from the sodium cation. KOH (model 1) requires 66.44% less Δ*E*_a_ than NaOH (model 2), considering vdW interactions in a configuration where the cation exhibits octahedral coordination and the hydroxide anions (OH^−^) exhibit tetrahedral coordination. This is due to the larger ionic radius of K^+^ (1.38 Å *vs.* 1.02 Å for Na^+^) results in a lower charge density and weaker electrostatic interactions with the aluminosilicate framework, facilitating structural disruption and making Al–O bonds easier to break. Additionally, the lower hydration energy of K^+^ allows for more effective ion exchange and interaction with the aluminosilicate network, further reducing the Δ*E*_a_ required for aluminate species dissolution in KOH compared to NaOH.

Models 3 and 4 represent KOH and NaOH with contribution of vdW interactions, respectively, where the cations are arranged in tetrahedral coordination, while the hydroxide anions exhibit trigonal planar coordination, as shown in Fig. 6 and 7. Fig. 6A shows that the potassium cation is optimized at a distance of 5.28 Å from oxygen (i), which 1.45 Å closer compared to the octahedral coordination shown in Fig. 4 due to less hydration shell around the cation. In contrast, an identical distance of 2.61 Å is observed for model 3C, when compared to model 1C. Lower Δ*E*_a_ for aluminate species dissolution were observed when the hydration shells surrounding KOH or NaOH were reduced, allowing for stronger cation–surface interactions and enhanced bond cleavage in the aluminosilicate network. Thus, the Δ*E*_a_ required for the configuration in model 3, using KOH as the activator, is 264.54% lower than that of model 1. Model 4, which employs NaOH activator, demands also 6.02% less Δ*E*_a_ than model 2. A similar trend was observed in the Δ*E*_a_ for KOH compared to NaOH with identical hydration shells around activators, with KOH requiring 470.21% less Δ*E*_a_. Fig. 8, representing model 5, highlights the contribution of water in Al bond breaking. It illustrates that a high energy of 3.157 eV is needed to reach the transition state.

### 3.2 Activation energy computation excluding vdW contributions

To fully understand the effect of excluding vdW interactions on the Δ*E*_a_ of a hydrolysis reaction and Δ*G*_reaction_ calculations, we analyze identical geometric structures depicted in Fig. 9–13. The vdW interactions between atoms and molecules are crucial in numerous chemical systems. They maintain the equilibrium condition with electrostatic and exchange–repulsion interactions, collectively shaping the system's behavior.^57^ The lower computed energy barrier required to reach the transition state, resulting in a more favorable Δ*E*_a_. Without vdW interactions, this interaction is absent, and the system requires more energy to overcome the barrier, leading to a higher (less favorable) Δ*E*_a_. For instance, Fig. 9, depicting model 1 without vdW interactions, shows a 29.46% higher Δ*E*_a_ for KOH compared to the identical geometric structure that includes vdW interactions, as illustrated in Fig. 4.

Fig. 10, illustrating model 2 without vdW interactions, reveals a 4.91% increase in the Δ*E*_a_ for KOH compared to the corresponding geometric structure that includes vdW interactions, as shown in Fig. 5. Likewise, Fig. 11, depicting model 3 without vdW interactions, shows a 6.03% rise in the Δ*E*_a_ for KOH relative to its vdW-inclusive counterpart in Fig. 6. Similarly, Fig. 12, representing model 4 without vdW interactions, exhibits a 9.46% higher Δ*E*_a_ for KOH compared to the identical structure incorporating vdW interactions, as illustrated in Fig. 7. Although vdW forces affect relative energy differences, such as Δ*E*_a_, they are generally weaker than covalent and electrostatic interactions. As a result, their inclusion primarily shifts energy levels rather than altering the molecular geometry, ultimately lowering the Δ*E*_a_. Thus, no significant changes in geometric structures were observed when vdW interactions were included, compared to the identical geometries obtained without vdW interactions.

## 4. Discussion

To provide a comprehensive understanding of species dissolution in MK, it is essential to compare aluminate species examined in this study with Si dissolution.^21^Fig. 14 summarizes the computed Δ*E*_a_ for aluminate species dissolution in this study, considering scenarios with and without vdW interactions and different hydration shell geometries around Na^+^, K^+^, and OH^−^. Table 2, adapted from recent study,^21^ provides the activation energies of SiO_4_^4−^ dissolution, including the effects of KOH and NaOH activators, both with and without vdW contributions for different simulation models. To ensure an accurate comparison between aluminate species and SiO_4_^4−^ dissolution, the A0S1 simulation model was selected for SiO_4_^4−^ dissolution. This is because A0S1 represents the dissolution of Si tetrahedral monomer, where the product geometry from the S0S0 model is reused as the reactant geometry in A0S1. The dissolution process involves the protonation of the bridging oxygen, which is ionically bonded to the neighboring aluminum cation, facilitating the dissolution of the silicate tetrahedra. Although the dissolution of aluminate species exhibited a low Δ*E*_a_ of less than 2 eV for all models with KOH and NaOH activators (Table 3), the dissolution of SiO_4_^4−^ in the presence of a NaOH activator without vdW contribution requires a Δ*E*_a_ exceeding 3 eV at the transition state. To compare the Δ*E*_a_ for SiO_4_^4−^ dissolution (Table 2), 1.462 eV is required in the presence of a KOH activator, while 1.949 eV is needed with a NaOH activator, both considering the contribution of vdW interactions. Accordingly, the Δ*E*_a_ for SiO_4_^4−^ dissolution is 418.4% and 21.2% higher than that of aluminate species in models 3 (0.282 eV) and 4 (1.608 eV), respectively, which incorporate vdW interactions in the presence of KOH and NaOH activators.

**Table 3:** Overview of Δ*E*_a_ and Δ*G*_reaction_ for all five presented model scenarios based on Fig. 1, both with and without vdW interaction contributions

Scenario (model)	1	2	3	4	5
Δ*E*_a_/Δ*G*_reaction_ including vdW (eV)	1.028/−1.325	1.711/−0.997	0.282/−1.627	1.608/−1.529	3.157/−0.883
Δ*E*_a_/Δ*G*_reaction_ excluding vdW (eV)	1.331/−1.257	1.795/−1.221	0.299/−1.667	1.760/−1.430	2.425/−0.977
Activator	KOH	NaOH	KOH	NaOH	Water

To summarize, Table 3 shows the detailed comparison of Δ*E*_a_ and Δ*G*_reaction_ across all five model scenarios based on Fig. 1, highlighting the variations in energy values with and without the inclusion of vdW interaction contributions. To place our findings in context, the study by Morrow *et al.*^58^ on Δ*E*_a_ for aluminate dissolution in aluminosilicate systems is also referenced here to enable a comprehensive comparison. Their work emphasizes the sensitivity of computed values to the choice of density functionals and basis sets, employing B3LYP, PBE1PBE, and M05-2X functionals in combination with the 6311+G(d,p) and MG3S basis sets, but without accounting for hydration shells or KOH and NaOH activators. In contrast, our study addresses this gap by incorporating diverse hydration shells and alkali activators, using the PBE(GGA) functional with plane-wave basis set. Table 4 presents a comparative summary of all Δ*E*_a_ values from both their study and this study. As a result, the differences in activation energy can be attributed to the use of different activators, the inclusion of hydration shell effects, and the choice of exchange–correlation functional in our calculations. However, for the KOH activator, our results align well with theirs, showing a lower activation energy that is consistent with their findings. Therefore, the most favorable computed Δ*E*_a_ in our study correspond to configurations involving vdW interactions with KOH (0.282 eV) and NaOH (1.608 eV) activators, where cations adopt tetrahedral coordination and hydroxide anions exhibit trigonal planar coordination during aluminate species dissolution, in good agreement with the study reported by Marcus.^41^

**Table 4:** The computed Δ*E*_a_ obtained using the B3LYP, PBE1PBE, and M05-2X functionals, as reported by Morrow *et al.*,^58^ are compared with those calculated using the PBE (GGA) functional in this study

	B3LYP		PBE1PBE		M05-2X		PBE(GGA)	
	6311+G(d,p)	MG3S	6311+G(d,p)	MG3S	6311+G(d,p)	MG3S	Plane-wave	
Δ*E*_a_ for Al dissolution(eV)	0.404	0.363	0.539	0.363	0.757	0.560	0.282	1.608

## 5. Conclusion

This paper aims to calculate the atomistic Δ*E*_a_ at the transition state for the hydrolysis reaction using MLFF based on DFT, employing IDM to determine the change in reaction enthalpy under far-from-equilibrium conditions. The computations, grounded in TST, aim to determine the atomistic Δ*E*_a_ for the dissolution of aluminate species, leading to the formation of aluminum hydroxide hydrate Al(OH)_3_(H_2_O)_3_ in an alkaline medium in MK, considering three activators: NaOH, KOH, and water. For this purpose, the study investigates both the presence and absence of vdW interactions, along with various geometric configurations of hydration shells surrounding cations (Na^+^, K^+^) and the hydroxide anion (OH^−^). The results showed that reduced hydration shells around KOH and NaOH led to lower Δ*E*_a_ for aluminate species dissolution by strengthening cation–surface interactions and facilitating bond cleavage within the aluminosilicate network. A higher activation energy (Δ*E*_a_) was observed for NaOH compared to KOH under identical hydration shell conditions, both with and without vdW interactions. In the absence of vdW interactions, the system experiences a higher energy barrier, making the activation process less favorable. Building upon earlier observations on SiO_4_^4−^ dissolution in MK, this study aims to bridge critical data gaps essential for understanding the dissolution of aluminate species in its 6-fold coordinated state (Al^VI^). The findings will complement all input data for microscopic forward dissolution rate calculations, which play a key role in the atomistic kMC upscaling methodology.

## Abbreviations

MK: metakaolin
DHX: dehydroxylation
SCM: supplementary cementitious material
Δ*E*_a_: activation energy
Δ*G*_reaction_: binding energy
kMC: kinetic Monte Carlo
MLFF: machine learning force fields
IDM: improved dimer method
NEB: nudged elastic band
IRC: intrinsic reaction coordinates
CGMC: coarse-grained Monte Carlo
DFT: density functional theory
MD: molecular dynamics
VDW: van der Waals
TST: transition state theory
VASP: Vienna *Ab initio* Simulation Package
GGA: generalized gradient approximation
PAW: projector augmented wave

## Data availability

All relevant data supporting this study are fully included in the Results and Discussion section of this article.

## Conflicts of interest

There are no conflicts to declare.
